# Genetic diversity analysis of fourteen geese breeds based on microsatellite genotyping technique

**DOI:** 10.5713/ajas.18.0589

**Published:** 2019-01-02

**Authors:** Hebatallah Abdel Moniem, Yang Yao Zong, Alwasella Abdallah, Guo-hong Chen

**Affiliations:** 1 Animal wealth development department, Faculty of veterinary medicine, Suez Canal University, Ismailia, 41611, Egypt; 2 Animal breeding and genetics department, Animal Science and Technology College, Yangzhou University, Yangzhou 225009, China; 3 Agricultural Research Corporation, Wad medani, 2111, Sudan

**Keywords:** Goose, Genetic Diversity, Population Structure, Microsatellite Marker

## Abstract

**Objective:**

This study aimed to measure genetic diversity and to determine the relationships among fourteen goose breeds.

**Methods:**

Microsatellite markers were isolated from the genomic DNA of geese based on previous literature. The DNA segments, including short tandem repeats, were tested for their diversity among fourteen populations of geese. The diversity was tested on both breeds and loci level and by mean of unweighted pair group method with arithmetic mean and structure program, phylogenetic tree and population structure were tested.

**Results:**

A total of 108 distinct alleles (1%) were observed across the fourteen breeds, with 36 out of the 108 alleles (33.2%) being unique to only one breed. Genetic parameters were measured per the 14 breeds and the 9 loci. Medium to high heterozygosity was reported with high effective numbers of alleles (Ne). Polymorphic information contents (PIC) of the screened loci was found to be highly polymorphic for eleven breeds; while 3 breeds were reported moderately polymorphic. Breeding coefficient (F_IS_) ranged from −0.033 to 0.358, and the pair wise genetic differentiation (F_ST_) ranged from 0.01 to 0.36 across the fourteen breeds; for the 9 loci observed and expected heterozygosity, and Ne were same as the breeds parameters, PIC of the screened loci reported 6 loci highly polymorphic and 3 loci to be medium polymorphic, and F_IS_ ranged from −0.113 to 0.368. In addition, genetic distance estimate revealed a close genetic distance between Canada goose and Hortobagy goose breeds by 0.04, and the highest distance was between Taihu goose and Graylag goose (anser anser) breed by 0.54.

**Conclusion:**

Cluster analyses were made, and they revealed that goose breeds had hybridized frequently, resulting in a loss of genetic distinctiveness for some breeds.

## INTRODUCTION

Geese play a minor role in meat and egg production compared to chicken worldwide. On the other hand, the nutrition values of protein, vitamin A, vitamin B, niacin and sugar content are higher in goose meat, than that in pork or mutton. The energy content is 30% to 63% greater than that of other poultry with the advantage of low-fat and cholesterol content [1,2]. Geese have many other economic benefits such as: their large body size, strong adaptability to extensive management, high reproduction rate, along with good disease resistance [3]. For all these reasons, geese production is of considerable importance and of great commercial interest.

In the past few decades, extensive production systems have been instituted by many countries aiming for high quality and quantity of meat as their primary breeding objective. As a direct result of these systems, some goose breeds with specific features either have declined or their breed characteristics diluted to improve their genetic admixture [4,5].

The evaluation of genetic variation can be done using several techniques. One of the most important techniques is the usage of microsatellites. Microsatellites have many advantages including their large number of polymorphisms, abundance, co-dominant inheritance, analytical simplicity, and transferability [6–8]. In recent years, microsatellite-based studies have been used for the genetic evaluation and mapping between local geese breeds in China, revealing that some geese breeds are at risk of becoming genetically homogenous [9]. The development of effective and appropriate breeding management practices is needed to maintain the genetic diversity and structure of these breeds [4,10,11]. Genetic structure of 14 grey goose breeds was studied using 31 microsatellite markers. A total of 25 were moderately polymorphic, and the phylogenetic tree was completed through analysis of unweighted pair group method with arithmetic mean (UPGMA) revealing three main branches, two for Chinese breeds and one for the Yili goose (Yi) breed [12].

In recent studies the genetic diversity among geese populations in Taiwan were evaluated along with industrial white Rom farms revealed unified genetic structures in their breeders, ensuring more stable and better performing populations. However, Chinese breeds raised at private farms revealed an uneven structure, indicating that breeding management requires urgent care to ensure stable production, maintain genetic resources, and develop hybrid geese for better meat quality [13].

The mitochondrial DNA control region sequence variation of domestic geese was analyzed to evaluate the main matrilineal components and their phylogenetic relationships. Our results supported that Chinese domestic goose breeds (except the Yi breed) originated from the Swan goose (Anser cygnoides) (Sw); while European goose breeds originated from Graylag goose (Anser anser) (Gray) [13,14].

In the present study, we used 14 goose breeds. They were classified according to their geographical distribution into: four Chinese breed (Yangzhou, Shitou, Yili, and Taihu); five Europeans (Landes, Roman, Leime, Carolas, and Hortobagy); 2 African Egyptian breeds; 2 wild (graylag and white swan and one American breed [Canada]). We evaluated both the genetic diversity and genetic relationship between these populations. In order to give recent information about genetic diversity among, and within these breeds to optimize the utilization of goose genetic resources, and permit efficient genetic improvement for both production and conservation needs.

## MATERIALS AND METHODS

### Population DNA samples

DNA samples [15] were obtained from 599 unrelated individuals representing 12 domestic and 2 wild geese populations; described as follow: 70 samples Landes goose (Land), 30 Roman goose (Rom), 30 Leime goose (Leim), 75 Yangzhou goose (Yang), 50 Shitou goose (Shi), 65 Yili goose (Yi), 48 Carlos goose (Ca), 48 Taihu goose (Tai), 30 Canada goose (Can), 30 Graylag goose (Gray), 30 Swan goose (Sw), 68 Hortobagy goose (Hort), 60 Egyptian goose (black variety) (Egy B), and 60 Egyptian goose (grey variety) (Egy G).

### Microsatellite sites selection in goose breeds

Nine species-specific microsatellite markers, isolated from geese, were chosen from GenBank and related articles [12,13] and used for geese breed genotyping. The primers were synthesized by Thermo Fisher Scientific Inc., Shanghai, China. Polymerase chain reaction (PCR) amplification was carried out in a 20 μL mixture containing 2 μL of 10×PCR (Mg^+2^) buffer, 2 μL of 10 mmol/L dNTPs, 1 μL of 10 μmol/μL forward primers, 1 μL of 10 μmol/μL reverse primers, 0.2 μL of 5.0 U/μL Taq DNA polymerase, and 1 μL of 100 ng/μL DNA template, and completed by adding 12.8 μL of double distilled water. After a denaturing step for 5 min at 95°C, samples were processed through 35 cycles of 45 s at 94°C, 45 s at an optimal annealing temperature (55°C to 64°C), and 45 s at 72°C. The last elongation step was extended to 10 min at 72°C, and preserved at 4°C as shown in (Table 1).

### Genotype of individuals and statistical analysis

After electrophoresis, the 9 fluorescent microsatellite primers were mixed according to their groups as shown in Table 1. DNA Analyzer fluorescence was measured by automatic sequencing of PCR products of short tandem repeat type. The PCR products were sent to Beijing New Industry Biotechnology Co., Ltd. (Beijing, China) (TSIGNKE). The independent documents were automatically generated by GeneMapper4.0 software containing fragment length, height of peak, and size of peak area.

The allelic data obtained through individual genotyping were analyzed by using different analytical software. MS toolkit (http://dscar.gene.ie-tcd./microsatellitetoolkit/m) program [16] was implemented to calculate allelic frequency, expected and observed heterozygosity (He and Ho) and polymorphic information content (PIC).

The indices of inbreeding (F_IS_) and genetic differentiation (F_ST_) were analyzed by FSTAT Weir [17]. Structure software was used for clustering analysis [18]. The Markov chain Monte Carlo procedure was used and 10 independent runs of each K were implemented with 1×10^6^ iterations after a burn-in period of 1×10^5^ iterations for 14 populations. The most likely number of populations (K) was determined according to the procedure explained by [19]; Dispan Procedures [20] and UPGMA to measure standard genetic distance, and genetic distance (DA) and Xlstat to construct Dendrograms of relationships based on these two kinds of genetic distances. Effective numbers of alleles (Ne) was calculated according to the equation stated by [21].

## RESULTS

The present study assessed the level of genetic diversity and the population structure of different geese breeds; genetic diversity was measured in terms of allelic frequency, Ho and He, PIC, Ne and inbreeding coefficient (F_IS_) over the fourteen breeds and the nine microsatellite loci.

### Allele frequency

The data set was analyzed to calculate allelic frequency, and to determine the presence of private alleles. A total of 108 distinct alleles (1%) were observed across the fourteen breeds, with 36 out of the 108 alleles (33.2%) unique to only one breed as shown in Table 2. With the highest 32% (Land at 152 bp) and the lowest 0.6% (Yang at 146 bp). Meanwhile, one locus G10 and four geese breeds Rom, Leim, Shi, and Egy B did not have any unique alleles.

**Figure 1:**
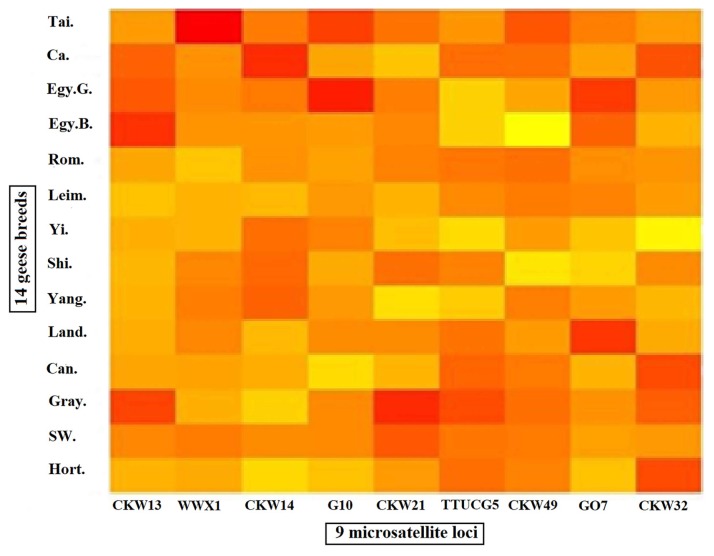
Heat map representing allele frequency for 9 loci in all 14 population as the gradual increase in color density from lighter color 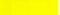 to darker color 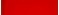 means the increase in the allele frequency as the red color, which indicates the highest value of allele frequency.

MATLAB software [22] was used to provide a platform for data visualization and construction of heat map representing allele frequency for all 9 loci in the 14 populations as presented in Figure 1. The interpretation of the color intensity was as follows: the lighter color means low frequency (as light yellow mean 0%), the gradual increase in color density means the increase in the allele frequency until reaching a red color, which indicates the highest value of allele frequency.

### Expected and observed heterozygosity, effective numbers of alleles, polymorphic information content and F_IS_ estimates

*Goose populations*: Genetic parameters were calculated for the fourteen geese breeds as shown in Table 3. Moderate to high level of average genetic diversity for both He and Ho were recorded; He ranged from 0.482 (Hort) to 0.69 (Sw and Gray), while Ho ranged from 0.345 (Hort) to 0.65 (Can). The PIC were found to be highly polymorphic for eleven breeds, while Hort, Can, and Tai. Were reported to be moderately polymorphic. The Ne ranged from 2.5 (Gray) to 3.39 (Yi). Breeding coefficient (F_IS_) revealed statistically significant differentiation (p<0.01) among the studied populations. The mean values of F_IS_ ranged from −0.033 (Ca) to 0.358 (Land).

*Microsatellite loci*: Same parameters were calculated per the nine loci as shown in Table 4. Low to high level of average expected heterozygosity (Exp He) and observed heterozygosity (Obs He) were recorded; He ranged from 0.28 (CKW13) to 0.848 (TTUCG5). Ho ranged from 0.26 (CKW13) to 0.6 (CKW21). PIC across the screened loci reported 6 loci highly polymorphic (PIC>0.5) for G1O, CKW21, TTUCG5, CKW49, GO7, and CKW32 and 3 loci reported medium polymorphic (PIC>0.25) for WWX1, CKW14, and CKW13. The Ne ranged from 1.39 (CKW13) to 6.294 (TTUCG5). The F_IS_ revealed statistically significant differentiation (p<0.01) among the nine loci and ranged from −0.113 (CKW14) to 0.368 (GO7).

### Genetic differentiation (F_ST_), genetic distance, and phylogenetic tree

*Genetic differentiation (F_ST_)*: The values of genetic differentiation (F_ST_) for each tested population pair are sum-marized in Table 5. It ranged from 0.01 (between Can and Hort) to 0.36 (between Hort and Egy B); and reported to be highly significant (p<0.01).

**Figure 2:**
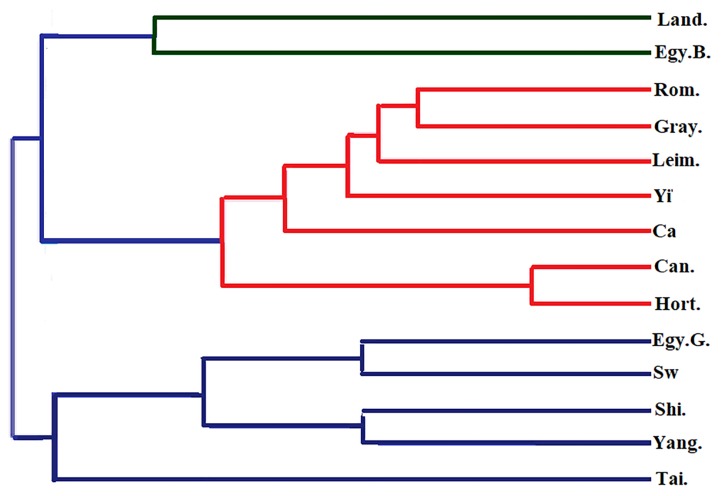
Unweighted pair group method with arithmetic mean (UPGMA) dendrogram based on genetic distance deriving from analysis of 9 microsatellite loci for the wild and hatchery populations of 14 geese breeds, which were divided into two clusters, one cluster contained breeds from Chinese origin Yangzhou goose (Yang), Shitou goose (Shi) and Taihu goose (Tai), along with swan (Sw) and Egyptian goose (grey variety) (Egy G) and the second cluster was subdivided into two clades first one contained Roman goose (Rom), Leime goose (Leim), Yili goose (Yi), Carlos goose (Ca), Canada goose (Can), Graylag goose (Gray) and Hortobagy goose (Hort), while second clade contained Egyptian goose (black variety) (Egy B), and Landes goose (Land).

*Genetic distance and phylogenetic tree*: The Nei’s [23] DA was calculated to analyze the distance between groups, using Dispan Software, as shown in Table 5. It ranged from 0.04 (between Can and Hort) to 0.54 (between Tai and Gray). Later on, the DA matrix of these populations was used to build phylogenetic trees by mean of the UPGMA. Using this technique as shown in Figure 2 the 14 geese breeds were divided into two clusters, one cluster contained breeds from Chinese origin (Tai, Yang, and Shi), along with Sw and Egy G breed and the second cluster was subdivided into two clades first one contained Hort, Can, Caro, Yi, Leim, Gray, and Rom while second clade contained Egy B and Land geese breed.

### Clustering

**Figure 3:**
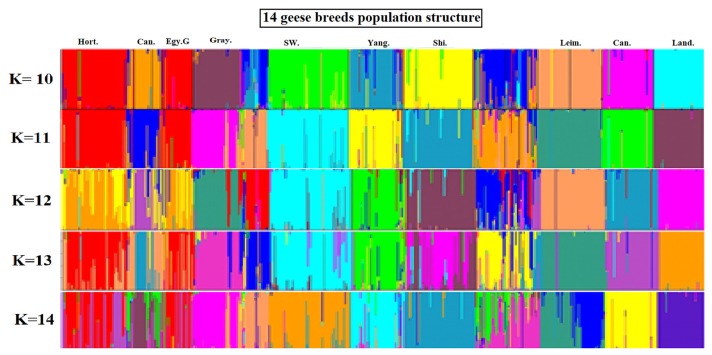
Structural analyses of goose populations. Each genotyped goose is represented by a single vertical line divided into K colors, where K is the number of clusters assumed in each structure analysis. Each vertical bar represents an individual goose. The colors on a vertical bar represent the probability that an individual belongs to that cluster. The Clustering diagrams of 14 goose breeds obtained from K = 10 to K = 14 using Q matrices of runs with best similarities When K = 10 there was a clear clustering for Hort, Can, Egy G, Gray, Sw, Yang, Shi, Leim, Ca, and Land. K = 11 was the same as K = 10 except for more interference between the breeds. K = 12 the clusters with clear identification were Gray, Sw, Yang, Shi, Leim, Ca, and Land. K = 13 can see clusters for only Gray, Sw, Yang, Leim, Ca, and Land. K = 14 the clearest clusters were for Gray, Sw, Yang, Shi, Ca, and Land. Hort, Hortobagy goose; Can, Canada goose; Egy G, Egyptian goose (grey variety); Gray, Graylag goose; Sw, along with swan; Yang, Yangzhou goose; Shi, Shitou goose; Leim, Leime goose; Ca, Carlos goose; Land, Landes goose.

The STRUCTURE software [18] program using Bayesian model-based clustering algorithms of multi-locus genotypes was utilized to assign individuals into populations via estimated individual admixture proportions and to infer the number of populations (K) for a given sample. The results from the analysis of all the populations for K = 10 to K = 14 are shown in (Figure 3). Some groups revealed a visual cluster but with interference of other groups.

When K = 10 there was a clear clustering for Hort, Can, Egy G, Gray, Sw, Yang, Shi, Leim, Ca, and Land. K = 11 was the same as K = 10 except for more interference between the breeds. K = 12 the clusters with clear identification were Gray, Sw, Yang, Shi, Leim, Ca, and Land. K = 13 can see clusters for only Gray, Sw, Yang, Leim, Ca, and Land. K = 14 the clearest clusters were for Gray, Sw, Yang, Shi, Ca, and Land.

## DISCUSSION

It is difficult and time-consuming to distinguish indigenous goose breeds on morphological characteristics alone, for this reason it is important to develop molecular markers to aid in goose breeds identification. This will also help in designing an effective breeding program to improve productivity of these breeds, and protect them from becoming extinct.

### Allele frequency

Allele frequency is defined as the relative frequency of an allele at a particular locus in a population, expressed as a fraction or percentage [24]. The change in allele frequencies that occurs over time within a population leads to genetic diversity. It is the base from which the other genetic parameters can be determined. Presence of private allele can be used as a tool to identify different goose breeds in agreement with [9,12–14].

### Expected and observed heterozygosity, effective numbers of alleles, polymorphic information content and F_IS_ estimates

Heterozygosity, reflects genetic variation in a tested locus among a population. High heterozygosity indicates low genetic uniformity thus high genetic diversity. The mean heterozygosity across all 14 populations and 9 loci in the present work ranged from medium at Hort 0.48 to high at the two wild breeds Sw and Gray 0.69; same as per loci medium at CKW13 0.28±0.12 to high at TTUCG5 0.84±0.03. Medium heterozygosity might be due to inbreeding in population because of the relatively small group in a breeding farm.

On the other hand, high heterozygosity was attributed to direct result of a breeding program based on selection to improve the genetic admixture for some breeds [4,5,25]. Observations of excess heterozygosity are not uncommon in geese, this was consistent with [12,26].

The PIC is a good index for gene fragment polymorphism. The PIC index can be used to evaluate the level of gene variation: when PIC<0.25, the locus has low polymorphism; when PIC>0.5, the locus has high polymorphism; and when PIC ranged between 0.25 and 0.5, the locus has intermediate polymorphism [27]. The PIC were found to be highly polymorphic for eleven breeds; while Hort, Can, and Tai were reported to be moderately polymorphic. In the same context, PIC reported CKW21, G10, TTUCG5, CKW49, G07, and CKW32 of high polymorphism and WWX1, CKW13, and CKW14 of medium polymorphism. This is in consistent with the sampling strategy to fully reflect the population genetic diversity information of the 14 populations. This also is in agreement with [10–14].

The effective population size (Ne) is the number of individuals in the idealized Wright Fisher [28] population that retains the same amount of genetic variation and experiences equally much genetic drift as an actual population irrespective of census size. The high Ne decreases genetic drift, which in turn increases heterozygosity. As mentioned by [1,10].

Population analysis F-statistics are a way of partitioning variances in gene frequencies among subpopulations by using ratios of different variances [29]. The relatively low but positive F_IS_ average, might indicate non-random mating, also these examined loci might be under morphological or productive traits of selective interest. Moreover, F_IS_ is used to obtain a deeper insight to appraise the degree of in-breeding and endangerment potentiality and is considered as an important tool to judge the conservation priority [30]. Accordingly, when F_IS_ is less than 0.05, the breeds are not in danger as in case of Ca breed; between 0.05 to 0.15, they are potentially endangered; between 0.15 to 0.25, they are minimally endangered as in case of Sw, Gray, Leim, Egy G, Egy B, Can, and Tai, breeds; between 0.25 to 0.40, they are endangered as in case of Hort, Rom, Yang, Shi, Yi, and Land breeds; and more than 0.40, they are critically endangered [31]. The F_IS_ results could be related to different factors such as population sub structuring or recent population growth [29]. These findings were the same as [9,12–14].

### Genetic differentiation, genetic distance, and phylogenetic tree

*Genetic differentiation (F_ST_)*: The pairwise genetic differentiation (F_ST_) is a measure of population differentiation due to genetic structure it ranges from 0 to 1. When F_ST_ = 0, there is no differentiation between the subpopulation. When F_ST_ = 1, all the alleles in the subpopulation are different [24,31]. The F_ST_ value across the 14 studied population showed low (0.01) to moderate mean (0.36) indicating that there is genetic differentiation among the 14 breeds for these values as F_ST_ was higher than 0.25 [29], this was recorded in both Egyptian breeds. While in Tai breed, this came along with a heterozygosity record indicating there is some migration occurring; which is an important mechanism for transferring genetic diversity among populations. This may result in a change in allele frequencies affecting the distribution of genetic diversity within the populations, due to new genetic variants be added to the established gene pool of a particular population. This comes in agreement with [9,12,24,30,31].

*Genetic distance and phylogenetic tree*: The project of breed protection and a plan of breeding can be made by analyzing genetic distance. Genetic distance should be an index for group structure and breeds diversity when breed conservation decisions are being made. Microsatellite allelic gene frequency analysis was one of the best methods at present, as it can reflect the time of diversity as well as genetics and variation among breeds [24].

A variety of genetic distances are currently available, but Nei DA is the most commonly used [23]. The DA was estimated for the 14 studied geese populations across the 9 microsatellite loci. The closest pairwise was recorded between Can and Hort (0.04) and this was supported by clustering in the neighbor-joining phylogenetic tree (Figure 2). The close relationship between Can and Hort can be attributed to the migrating nature of both breeds with the possibility of hybridization between both. Also the close genetic distance between Chinese breeds (Yang, Shi, and Tai) indicates the production system used by China aimed for high quality and quantity of meat [4,5]. On contrary, the widest genetic distance was recorded between Tai and Gray breeds was 0.54.

The clustering based on UPGMA and Nei’s distance is the best method for analyzing genetic diversity in different breed population. Nei [23] discovered that the correct topological tree is more effectively obtained from DA than other genetic distances, when he adopted infinite-allele model for computer simulation; 14 goose breeds were clustered into 2 main branches, which later on divided to four groups. This process reflected their relationship on geographical distribution and origin to some degree. The same findings were found in the previous literature suggesting that Chinese goose breeds (Tai, Yang, and Shi) were derived from the Sw, except for Yi breed, and European goose breeds (Hort, Can, Rom, and Leim) along with Yi breed were derived from the Gray. This assumption is based on morphology and plumage patterns; while for Yi breed this can be attributed to being in conservation zone and under the management of breeding program to improve its low performance [12,14].

### Clustering

The Structure program is based on the Bayesian probability theory and uses the Markov-Monte Carlo simulation algorithm. When the program runs, a mixed model is used to set the number of classification K of the detected population. All individuals can be divided to reflect the genetic structure of the population, especially for the population genetic structure and individual differentiation, migration and other aspects of the study. The structure program can also infer individuals with complex genetic background or migrating individuals in a population based on the number of individual alleles [19]. The attribution judgment of individuals in a group is not possible by ordinary genetic distance-based clustering methods. In this study, 14 groups were analyzed using the Structure program, the population structure was inferred, the accurate population structure map was obtained, K = 10, K = 11, K = 12, K = 13, and K = 14. These values are consistent with the results calculated by genetic distance and phylogenetic tree structure although it was reported that the most probable population structure was at K = 10 (Figure 3), at which the Chinese goose breeds (Tai, Yang, Shi, and Yi) were clustered together forming admixed mosaic cluster. A probable explanation for Yi breed to be interfered with other lines from other populations is that the breed is in conservation zone and under the management of breeding program to improve its low performance [14]; this comes in agreement with the other parameters of the breed (He 0.63 and PIC 0.59). The high genetic admixture and migrations between Egy B and interference from other Chinese and European breeds strains could contribute them forming the admixed mosaic cluster with no clear cluster for the breed.

### Conclusions

Microsatellite markers are the more credible tool for the research of breed origins. This is due to the evolution of breeds in nature, and artificial selection had a little effect on the structure of Microsatellite locus. The clustering results support the hypothesis that geographical distance is an important factor influencing the genetic relatedness of populations’ and provided some useful data for evaluation of breeding programs results.

## Figures and Tables

**Table 1:** Characterization of the nine microsatellites loci used for short tandem repeat genotype

Group number	Locus	Primer (5′→3′)	Repeat motif	Size of alleles (bp)	Annealing temperature (°C)
1	WWX1	F:ATGGATGCTAACAAACACTC	(CCAAT)4	127–152	59
		R:GTACAAAGGTCATGGAGAAG			
	CKW13	F:AGGCTGAGGTGGGAGAATTTAT	(AAAC)5	150–154	53.4
		R: TTCTTCCACTTCTCCCAAAGAA			
2	CKW14	F:AACTGATCCGGCAGAAAACTAA	(CCT)5	219–223	60
		R:ACTTAGCATGCAGCTTCACAAA			
	CKW21	F:CCCAGAACAGTGCTAGAAGAGG	(TTA)10	236–272	60
		R:AGCGAGTCACTCCAGTACCTTC			
	G10	F:ACGCTGGCAGATCTTGATGTC	(CT)7	156–170	57.1
		R: TTAAAGCCTGTTCTCTGTAC			
3	TTUCG5	F:GGGTGTTTTCCAACTCAG	(TCTAT)8	175–305	53.7
		R:CACTTTCCTTACCTCATCTT			
	CKW49	F:TGAACACACATGCAGACTGG	(CA)10	188–208	62.1
		R: TTTGCGAGACAGAGCCTTTT			
4	G07	F:ACAGGTGATGCTATTATTACG	(AT)12	144–172	55
		R: CATTCCCTAGGAACAACCTGC			
	CKW32	F:CAGTGCAAGTTCACCCACAG	(AAAAT) 7	155–235	55.4
		R: TCGAGAGCACTCCATTTTGA			

**Table 2:** Mean number of alleles per locus for private allele across 14 geese population

Geese breeds	CKW13	WWX1	CKW14	CKW21	TTUCG5	CKW49	GO7	CKW32
Hort					0.74/160	0.73/222		
Sw	2.94/153				14.7/170			
Gray			6.25/227	18.75/230			16.3/173	
Can				1.67/246				1.67/156
Land			2.86/216				32/152	
Yang							0.6/146	
Yi				0.76/265				
Egy G							3.33/148	
Ca							2.08/172	1.03/230
Tai		1.04/122						1.04/225

Hort, Hortobagy goose; Sw, along with swan; Gray, Graylag goose; Can, Canada goose; Land, Landes goose; Yang, Yangzhou goose; Yi, Yili goose; Egy G, Egyptian goose (grey variety); Ca, Carlos goose; Tai, Taihu goose.

**Table 3:** Mean estimates of expected (He), observed (Ho) heterozygosity, effective numbers of alleles (Ne), polymorphic information content (PIC), and F_IS_ estimates per breed

Geese breeds	He	Ho	PIC	Ne	FIS
Hort	0.482±0.252	0.345±0.224	0.449±0.24	2.54±1.533	0.287**
Sw	0.69±0.14	0.6±0.1	0.73±0.17	3.66±1.9	0.179**
Gray	0.69±0.17	0.5±0.2	0.64±0.15	2.5±1.45	0.17**
Can	0.51±0.225	0.4±0.272	0.464±0.221	2.609±1.58	0.216**
Land	0.64±0.173	0.414±0.121	0.603±0.172	3.366±1.445	0.358**
Yang	0.61±0.179	0.422±0.153	0.57±0.172	3.042±1.32	0.309**
Shi	0.59±0.178	0.429±.163	0.549±0.175	2.948±1.384	0.286**
Yi	0.63±0.199	0.463±0.145	0.593±0.195	3.39±1.67	0.263**
Leim	0.61±0.173	0.507±0.129	0.557±0.173	3.114±1.781	0.168**
Rom	0.61±0.161	0.437±0.144	0.557±0.156	2.908±1.193	0.286**
Egy B	0.61±0.196	0.463±0.141	0.567±0.196	3.333±2.036	0.249**
Egy G	0.62±0.132	0.5±0.14	0.564±0.145	3±1.408	0.199**
Ca	0.63±0.121	0.65±0.11	0.572±0.138	3.066±1.468	−0.033**
Tai	0.522±0.228	0.394±0.22	0.488±0.221	2.73±1.614	0.248**

Hort, Hortobagy goose; Sw, along with swan; Gray, Graylag goose; Can, Canada goose; Land, Landes goose; Yang, Yangzhou goose; Yi, Yili goose; Leim, Leime goose; Rom, Roman goose; Egy B, Egyptian goose (black variety); Egy G, Egyptian goose (grey variety); Ca, Carlos goose; Tai, Taihu goose.

Mean estimates from jack-knife over loci, standard deviations are given in parentheses;

** p<0.01.

**Table 4:** Mean estimates of expected (He), observed (Ho) heterozygosity, effective numbers of alleles (Ne), polymorphic information content (PIC) and F_IS_ estimates per selected fluorescence primer loci

Loci	He (Exp He)	Ho (Obs He)	PIC	Ne	F_IS_ (Small F)
CKW13	0.28±0.12	0.26±0.1	0.26±0.1	1.4±0.2	0.065**
WWX1	0.49±0.11	0.38±0.2	0.46±0.2	1.9±0.4	0.252**
CKW14	0.51±0.14	0.56±0.12	0.45±0.12	2.1±0.6	−0.113**
G1O	0.68±0.1	0.46±0.09	0.62±0.09	3.36±1.1	0.288**
CKW21	0.79±0.04	0.6±0.05	0.75±0.05	4.48±1.02	0.262**
TTUCG5	0.84±0.03	0.59±0.04	0.82±0.04	5.86±0.9	0.312**
CKW49	0.61±0.15	0.43±0.16	0.56±0.16	2.6±1.03	0.346**
GO7	0.65±0.13	0.43±0.14	0.61±0.14	3±0.9	0.368**
CKW32	0.58±0.11	0.47±0.15	0.55±0.15	2.45±0.6	0.222**

**Table 5:** The Nei’s genetic distance (upper diagonal) and genetic differentiation F_ST_ between different populations (lower diagonal)

Geese breeds	Hort	Sw	Gray	Can	Land	Yang	Shi	Yi	Leim	Rom	Egy B	Egy G	Ca	Tai
Hort	-	0.3	0.25	0.04	0.24	0.32	0.18	0.28	0.17	0.21	0.44	0.36	0.21	0.44
Sw	0.19	-	0.3	0.34	0.28	0.39	0.29	0.3	0.31	0.25	0.36	0.37	0.36	0.36
Gray	0.17	0.07	-	0.29	0.37	0.42	0.3	0.38	0.30	0.35	0.41	0.39	0.41	0.54
Can	0.01	0.18	0.17	-	0.27	0.31	0.23	0.32	0.19	0.22	0.47	0.39	0.2	0.46
Land	0.18	0.12	0.14	0.17	-	0.25	0.27	0.28	0.17	0.15	0.34	0.31	0.18	0.29
Yang	0.24	0.18	0.17	0.21	0.15	-	0.16	0.17	0.21	0.24	0.27	0.25	0.23	0.19
Shi	0.18	0.14	0.13	0.18	0.10	0.14	-	0.21	0.18	0.2	0.29	0.29	0.24	0.18
Yi	0.16	0.12	0.14	0.16	0.14	0.16	0.12	-	0.24	0.29	0.26	0.24	0.33	0.36
Leim	0.13	0.12	0.08	0.12	0.07	0.13	0.12	0.12	-	0.13	0.29	0.23	0.21	0.36
Rom	0.16	0.11	0.14	0.15	0.07	0.15	0.12	0.13	0.05	-	0.38	0.3	0.18	0.27
Egy B	0.36	0.2	0.19	0.33	0.23	0.22	0.22	0.24	0.21	0.24	-	0.18	0.41	0.39
Egy G	0.27	0.17	0.15	0.25	0.2	0.19	0.19	0.21	0.14	0.19	0.11	-	0.39	0.33
Ca	0.18	0.18	0.19	0.15	0.1	0.15	0.16	0.17	0.12	0.13	0.25	0.22	-	0.38
Tai	0.35	0.16	0.29	0.33	0.21	0.27	0.27	0.27	0.26	0.21	0.32	0.27	0.28	-

Hort, Hortobagy goose; Sw, along with swan; Gray, Graylag goose; Can, Canada goose; Land, Landes goose; Yang, Yangzhou goose; Yi, Yili goose; Leim, Leime goose; Rom, Roman goose; Egy B, Egyptian goose (black variety); Egy G, Egyptian goose (grey variety); Ca, Carlos goose; Tai, Taihu goose.
